# Effects of Aged Conditions on the Self-Healing Performance of Asphalt Mixtures: A Comparative Study of Long-Term and Short-Term Aging

**DOI:** 10.3390/polym17192678

**Published:** 2025-10-03

**Authors:** Zhenqing He, Anhua Xu, Aipeng Wang, Tengyu Zhu, Bowen Guan

**Affiliations:** 1 Key Laboratory of High Altitude and Mountain Passway Construction and Maintenance, Qinghai Vocational Technical University, Xining 810003, China; hezqing123@chd.edu.cn; 2 School of Materials Science and Engineering, Chang’an University, Xi’an 710061, China; 2022131068@chd.edu.cn (A.W.); k747493297@163.com (T.Z.)

**Keywords:** steel slag asphalt mixture (SSAM), aging, microwave heating, self-healing, digital image correlation (DIC)

## Abstract

This study investigates how short- and long-term aging affect the microwave self-healing of steel slag asphalt mixtures (SSAMs). Binder-level healing was tested using a dynamic shear rheometer (DSR), and mixture-level crack behavior was analyzed using beam bending tests (BBTs) and digital image correlation (DIC). Aging clearly reduced self-healing, with long-term aging causing the largest decline. Among the mixtures, OGFC-13 was most sensitive, while SMA-13 was least affected. Aging increased stiffness, reduced crack resistance, and shortened crack initiation time, leading to lower healing efficiency under microwave treatment. After heating, cracks propagated faster, indicating increased brittleness. These results quantify the impact of aging on performance and highlight the limitations of microwave repair, providing guidance for maintenance strategies and mixture design to improve long-term pavement performance.

## 1. Introduction

As one of the main pavement types, asphalt pavement is prone to early surface damage during long-term service due to environmental factors and traffic loads [1,2]. Timely heating repairs are crucial to extend its service life [3,4]. Microwave heating is an efficient and environmentally friendly method. It can rapidly and uniformly heat the asphalt, softening the binder and restoring its adhesive properties. Cracks, potholes, and other defects can be precisely repaired without large-scale excavation, minimizing traffic disruption [5]. However, service conditions, as well as the aging level, moisture content, and composition of the asphalt mixture, can affect microwave heating efficiency and repair quality [6,7,8,9]. Therefore, these factors must be considered when optimizing heating parameters and repair materials to ensure long-term durability.

Extensive research has investigated the microwave self-healing performance of asphalt mixtures under complex conditions. Zhang et al. [3] studied freeze–thaw cycles and microwave heating and found that repeated heating gradually reduces healing capacity. Jing et al. [10] showed that pores of different sizes contribute differently during damage and healing: small pores dominate during freeze–thaw, while both small and large pores affect healing. Wang et al. [11] examined steel slag asphalt mixtures (SSAMs) under combined salt corrosion and freeze–thaw, identifying 40 s as the optimal heating time. Other studies revealed that corrosion severely reduces healing [12] and that higher fatigue damage leads to slower recovery [13]. International studies, including Ghazali et al. [12] and Pasetto et al. [14], also highlighted the environmental and mechanical benefits of steel slag mixtures and their suitability for microwave repair.

Most previous studies focus on external factors such as freeze–thaw cycles or corrosion. The influence of asphalt aging on microwave healing is less understood. Aging, caused by UV exposure and thermal–oxidative reactions, changes asphalt chemistry, increases stiffness, and reduces molecular mobility, limiting binder flow and crack closure under microwave heating [15,16]. Although a few studies have examined aging effects, comprehensive assessments combining rheological tests, mixture-scale fracture tests, and full-field strain monitoring are rare.

Steel slag (SS), a by-product of steelmaking, is widely used in asphalt pavements due to its mechanical strength and wear resistance [17,18]. Rich in metal oxides, SS absorbs microwaves efficiently, making SSAM suitable for microwave repair [14]. Incorporating SS improves mixture strength, rutting resistance, and durability while promoting resource efficiency and environmental benefits [5,19].

This study fills the research gap by systematically investigating short-term and long-term aging effects on the microwave self-healing performance of SSAM. We first evaluate aged binders using rheological and fracture tests. Then, Digital Image Correlation (DIC) monitors crack initiation, propagation, and strain field evolution in SSAM before and after aging and microwave repair. Finally, the average crack propagation rate is measured to reveal how aging affects microwave-induced self-healing. The novelty lies in combining binder-scale (DSR and BBT) and mixture-scale (DIC) evaluations across three representative mixtures (AC-13, SMA-13, OGFC-13), providing a comprehensive multiscale understanding of aging effects.

## 2. Materials and Methods

### 2.1. Materials

The experiment utilized 70# base asphalt, coarse aggregate (limestone), fine aggregate (granite), steel slag, and filler, with their key properties listed in Table 1. The 70# base asphalt was supplied by Xi’an Petrochemical Co., Xi’an, China, while coarse and fine aggregates were obtained from Xi’an Quarry, Xi’an, China. Steel slag aggregate (SSA) replaced limestone aggregate in the 4.75–9.5 mm size range, with a replacement rate of 75% based on previous studies [11]. The SSA employed is a by-product of the basic oxygen furnace (BOF) steelmaking process from Xi’an Steel Plant, Xi’an, China, and had been stored for three years to ensure volumetric stability, during which the f-CaO content decreased by 2.2%. Filler was provided by Xi’an Filler Materials Co., Xi’an, China. The chemical composition of SSA is presented in Table 2.

### 2.2. Design of Asphalt Mixture Gradation

**Figure 1:**
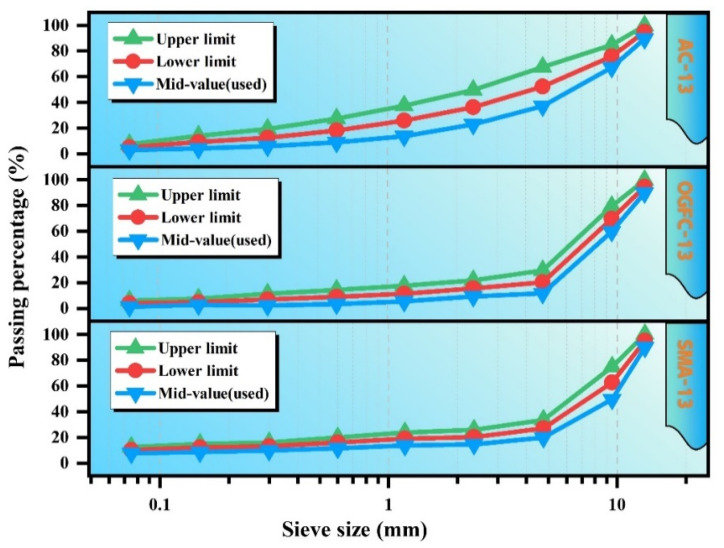
Gradation curves of SSAM.

This study designs three types of SSAM mixtures: AC-13, SMA-13, and OGFC-13. These widely used asphalt mixtures were selected to allow a comprehensive evaluation of their microwave self-healing performance after aging. The target gradations were determined based on the median values in accordance with relevant asphalt mixture gradation design standards [20]. The gradation curves are shown in Figure 1. Using the Marshall design method, the optimal asphalt-to-aggregate ratios were determined as 4.8% for AC-13, 5.8% for SMA-13, and 4.2% for OGFC-13.

### 2.3. Aging and Healing Tests of Asphalt

#### 2.3.1. Short-Term and Long-Term Aging

Short-term aging test is conducted according to the T0610 test method in the industry standard of China (JTG E20-2011E) [21], using a 52-LQ-615 asphalt rotary thin film oven (RTFO). Long-term aging was conducted following the T0630 method with a pressure aging vessel (PAV) [21].

#### 2.3.2. Dynamic Shear Rheometer (DSR) Test

**Figure 2:**
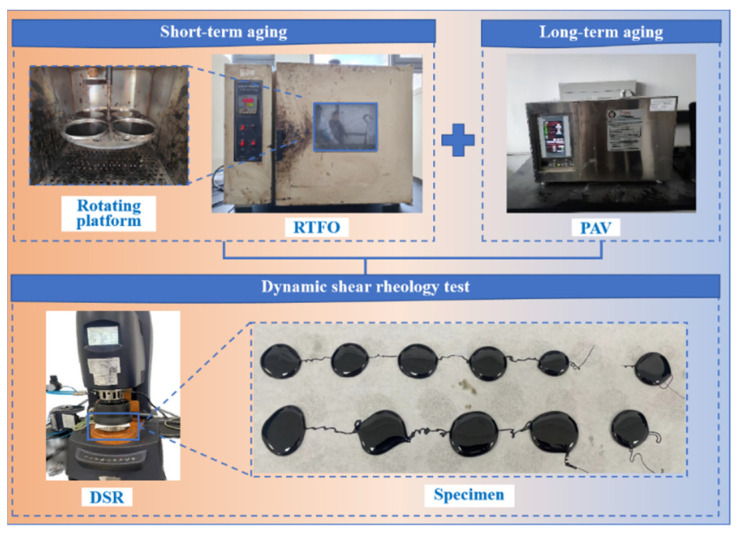
Aging and healing test of asphalt binders.

This study assesses the healing capacity of asphalt binders following short-term and long-term aging, as illustrated in Figure 2. A dynamic shear rheometer Discovery HR-20 (TA Instruments, New Castle, DE, USA) was used to conduct time-sweep tests with intermittent periods (loading–rest–loading) on both aged and unaged binders. The tests were carried out at 20 °C under strain-controlled cyclic loading with a frequency of 10 Hz. For each specimen, loading was applied until the shear complex modulus (G*) decreased to 60% of its initial value (Gi*). The loading was then stopped, and the specimens were allowed to heal for intervals ranging from 5 s to 2 h. After the rest period, the specimens were reloaded until failure occurred.

#### 2.3.3. Self-Healing Models

The performance degradation of SSAM due to aging is fundamentally attributed to the aging of the asphalt binder. This study adopts the Wool and O’Connor model to interpret asphalt binder healing from both mechanical and chemical perspectives [22]. Equation (1) represents the complete healing process of asphalt binders:

(1) $$R(t)=R_{0}+Kt^{1/4}\approx H(t)$$

In this equation,

*R(t)* indicates the total healing, which is derived from healing test data and depends on the rest time,

*R*_0_ represents the immediate healing resulting from the wetting of crack surfaces, which is influenced by the material’s adhesive properties,

*K* denotes the strength development rate driven by molecular diffusion across crack surfaces at a given temperature,

*t* represents the healing time.

The second component of the equation accounts for long-term healing, which is influenced by molecular diffusion at the crack surfaces and the length of the molecular chains [23,24].

The Wool and O’Connor models provide a reasonable framework for analyzing the self-healing behavior of asphalt mixtures. However, these models are based on idealized assumptions and may not fully capture the effects of aging, mixture heterogeneity, or varying environmental conditions, which could influence the actual healing efficiency.

#### 2.3.4. Healing Index

Based on time sweep tests with intermittent loading (loading–rest–loading), healing is quantified as a function of the rest period [25]. Equation (2) is used to calculate the healing index:

(2) $$H(t)=\frac{G_{r}^{*}-G_{L}^{*}}{G_{i}^{*}-G_{L}^{*}}$$

In this equation,

*H(t)* is the healing index;

$G_{i}^{*}$ is the initial modulus (MPa);

$G_{r}^{*}$ is the modulus after the rest period (MPa);

$G_{L}^{*}$ is the modulus after loading (MPa).

The healing index (*H(t)*), which is approximately equal to *R(t)*, is calculated for different rest periods and incorporated into the self-healing model (Equation (1)) to quantify both instantaneous and long-term healing.

### 2.4. Aging and Microwave Heating Self-Healing of SSAM

#### 2.4.1. Short-Term and Long-Term Aging

The SSAM mixtures were first prepared using fresh, unaged asphalt binders and aggregates. Short-term and long-term aging tests were then conducted following the T0734-2000 test method in the Chinese industry standard JTG E20-2011E [21].

#### 2.4.2. Microwave Self-Healing Performance Test

The asphalt beam bending test (BBT) was used in this study. Tests were conducted following the JTG E20-2011 specification [21], Test Method T0715. Beam specimens (250 × 30 × 35 mm) were subjected to three-point bending under displacement control at −10 °C to measure flexural strength and stiffness modulus. A UTM-25 multifunctional testing machine (IPC Global, Toorak, Australia) ensured accurate load and displacement control.

After testing, the fractured beams were dried, reassembled, and heated in a 700 W microwave at 2.45 GHz. Heating was applied in cycles of 40 s, followed by a 1 h rest, repeated four times. After microwave heating, the samples healed at room temperature for 24 h.

Following the healing period, the beams underwent a second three-point bending test to measure post-healing flexural strength, completing the damage-healing-damage cycle. Three replicates were tested to calculate the average flexural strength.

**Figure 3:**
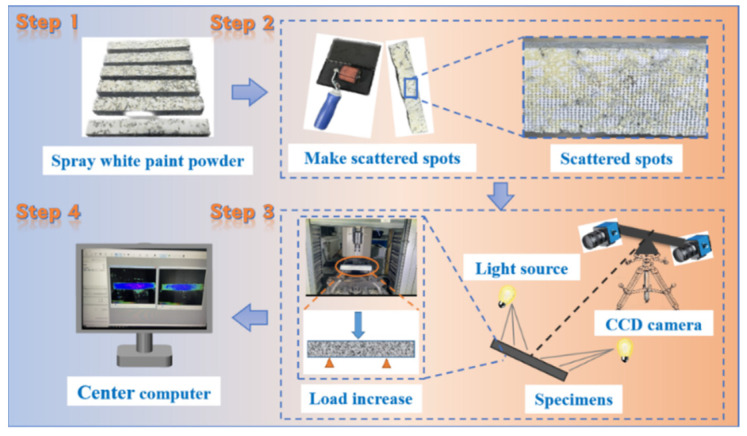
Process flow diagram for both the beam bending test and the DIC test.

A three-dimensional digital image correlation (DIC) system using VIC-3D provided full-field data on shape, displacement, and strain. Figure 3 shows the flowchart of the BBT and DIC testing procedure.

It should be noted that uneven distribution of microwave-absorbing materials may affect heating uniformity. Steel slag was the main microwave-absorbing component, but it is not the only mineral phase in the mixture. The spatial distribution of steel slag was not quantified, which may lead to local differences in heating efficiency. Infrared thermography could provide valuable insights into temperature uniformity and is recommended for future studies.

## 3. Results and Analysis

### 3.1. Influence of Aging on the Self-Healing Capability of Asphalt BINDER

Tables S1–S3 (Supplementary Materials) provide the full complex shear modulus (G*) values of asphalt under different rest periods and aging conditions. For clarity, only the key trends and average healing indices are discussed here.

For unaged asphalt, the initial modulus remains stable with increasing rest periods. Both the modulus after loading and the modulus after rest increase with longer rest periods, reflecting continuous healing. The healing index rises from 17% at 5 s rest to 51% at 60 s, with an average healing index of approximately 70.43%, consistent with previous findings [15].

For short-term aged asphalt, the modulus changes follow a similar trend, but the increase is smaller than that of unaged asphalt. The healing index increases from 7% at 5 s to 46% at 60 s, with an average healing index of 58.14%, showing a 12.29% reduction due to short-term aging.

For long-term aged asphalt, the modulus increases are further reduced. The healing index rises from 9% at 5 s to 37% at 60 s, with an average value of 42.96%, representing a 27.47% decrease compared to unaged asphalt. This confirms that long-term aging markedly diminishes self-healing capacity.

These results quantitatively demonstrate the effect of aging on binder-level healing. Notably, the slower recovery of modulus and lower healing index in aged binders indicate reduced mobility of asphalt molecules and hindered crack closure. Detailed numerical values are provided in Tables S1–S3 for reference.

**Figure 4:**
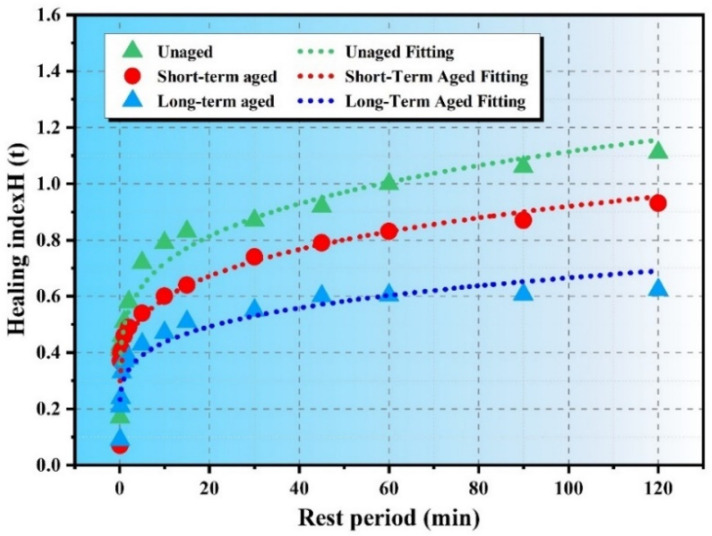
Healing models under different aging conditions.

Figure 4 shows how the healing index (H) of asphalt changes with the rest period (t) under different aging conditions. The results indicate that unaged asphalt heals better than short-term and long-term aged asphalt. As shown in the figure, the healing index decreases as aging increases. Over time, unaged asphalt maintains a higher healing index, indicating faster recovery from damage compared to aged asphalt. Similar trends were reported by Asadi et al. [26].

For aged asphalt, two distinct healing phases are observed. In the first 60 s of the rest period, the healing index increases rapidly, followed by a slower rise for the remainder of the period. This pattern is similar to that of unaged asphalt.

The healing indices under different aging conditions were fitted using Equation (1) to predict both instantaneous and long-term healing capacities. Table 3 summarizes the fitted curves and related parameters. The R^2^ values of all fitted curves exceed 0.87, indicating high model accuracy and suggesting that this physico-chemical model well describes the relationship between healing index and rest period [27]. In the model, R0 represents the instantaneous healing component, while K represents the long-term healing component. A higher K value indicates greater long-term healing capability. Overall, the fitting results reflect the impact of aging on asphalt self-healing: unaged asphalt shows the strongest self-healing ability, which gradually decreases with aging. Long-term aged asphalt exhibits a significant reduction in healing performance.

### 3.2. Influence of Aging on Microwave Heating Self-Healing of SSAM

#### 3.2.1. Asphalt Mixture Damage and Healing Effectiveness

Table 4, Table 5 and Table 6 present the results of three-point bending tests for three types of SSAM (AC-13, OGFC-13, SMA-13) under different aging conditions (unaged, short-term aged, long-term aged) before and after microwave heating self-healing. By comparing the performance changes in the mixtures after short-term and long-term aging, the impact of aging on different types of mixtures can be analyzed. Aging decreases the load-bearing capacity, bending strength, and ductility of mixtures, while increasing the stiffness modulus. Short-term aging leads to reductions in maximum load, maximum bending strength, and maximum strain for all three mixtures, with an increase in stiffness modulus, indicating that the mixtures become stiffer and less ductile. These properties further decline after long-term aging [28].

Among the mixtures, OGFC-13 shows the most significant degradation due to aging, followed by AC-13, and SMA-13 is the least affected. For OGFC-13, the maximum load decreases from 1.17 kN to 0.52 kN, bending strength from 8.9 MPa to 4.2 MPa, and bending strain from 3.26 × 10^−3^ to 1.04 × 10^−3^, representing reductions of approximately 55%, 53%, and 68%, respectively. This significant decrease is primarily due to the high air void content of OGFC, making it more susceptible to environmental factors. For AC-13, the reductions are about 48% in maximum load, 47% in bending strength, and 54% in bending strain. In contrast, SMA-13 experiences reductions of approximately 38%, 37%, and 57%, respectively, demonstrating the best aging resistance among the three types. This improved performance is attributed to SMA’s dense stone skeleton and optimized gradation design, which reduce the likelihood of aging-induced damage and, due to its higher asphalt content, provide better protection that slows down the aging process.

To evaluate the self-healing performance of SSAM, the maximum load before and after healing is compared. The healing rate is calculated by Equation (3):

(3) $$HI=\frac{F_{0}}{F_{1}}$$

In this equation,

*HI* represents the healing rate (%);

*F*_0_ represents the maximum load after healing (kN);

*F*_1_ represents the maximum load before healing (kN).

**Figure 5:**
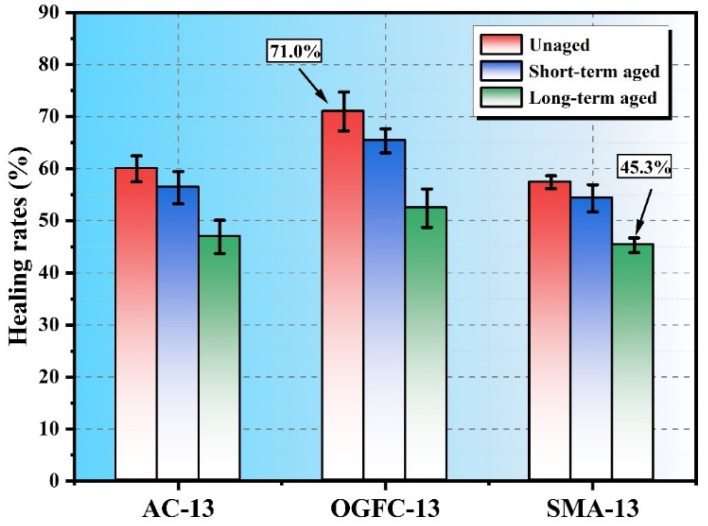
Self-healing performance of microwave-heated SSAM after aging. Error bars indicate standard deviation (*n* = 3).

Figure 5 illustrates the healing rates of three types of SSAM (AC-13, OGFC-13, SMA-13) under various aging conditions (unaged, short-term aged, and long-term aged). The results indicate that the healing rate decreases with increasing aging, demonstrating that the aging process reduces the self-healing capability of the mixtures. Specifically, the healing rate of AC-13 decreases from 60% for unaged asphalt to 46.9% for long-term aged asphalt. For OGFC-13, the healing rate drops from 71% to 52.4%, while SMA-13 shows a decrease from 57.4% to 45.3%. Short-term aging has a relatively minor effect on the healing rate, while long-term aging significantly impairs it. Among the mixtures, OGFC-13 consistently shows better self-healing performance across all aging stages. Although microwave heating combined with SS improves the heating and self-healing properties of the SSAM, the irreversible chemical changes caused by aging limit the extent to which performance can be restored [16].

#### 3.2.2. Crack Initiation Time and Crack Propagation Rate

**Figure 6:**
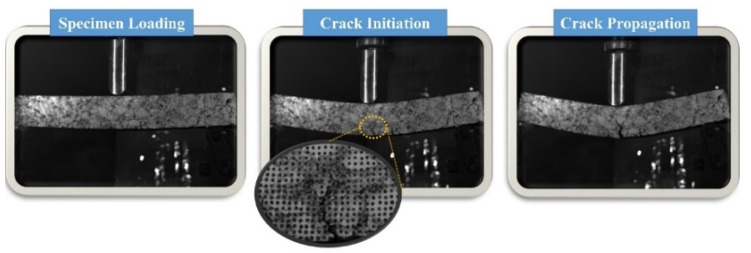
Crack initiation and propagation. Error bars indicate standard deviation (*n* = 3).

By observing the displacement or strain field data provided by the DIC system, the crack initiation zone can be identified at points of sudden changes in displacement or strain [29,30]. When a region experiences a sudden and sustained increase in displacement or strain, it is considered the onset of a crack. The DIC system can directly visualize crack formation and record the time of crack initiation. Figure 6 illustrates the entire process of crack formation and propagation under load. Typically, cracks initiate at the bottom and propagate upwards, circumventing the aggregate within the mixture, leading to varying crack propagation patterns and times among different specimens [31]. The DIC system provides images and timestamps when the maximum crack length is reached, allowing for the calculation of the average crack propagation rate. The specific method involves converting the original images into grayscale, applying gain reduction and noise filtering, and using the OTSU algorithm for crack binarization [32]. MATLAB software (MATLAB2025a) is then utilized to calculate the crack length, and the average crack propagation rate is computed using Equation (4):

(4) $$V=\frac{L}{t}$$

In this equation,

*V is* the average crack propagation rate (mm/s);

*L* is the crack length (mm);

*t* is the time of crack development (s).

**Figure 7:**
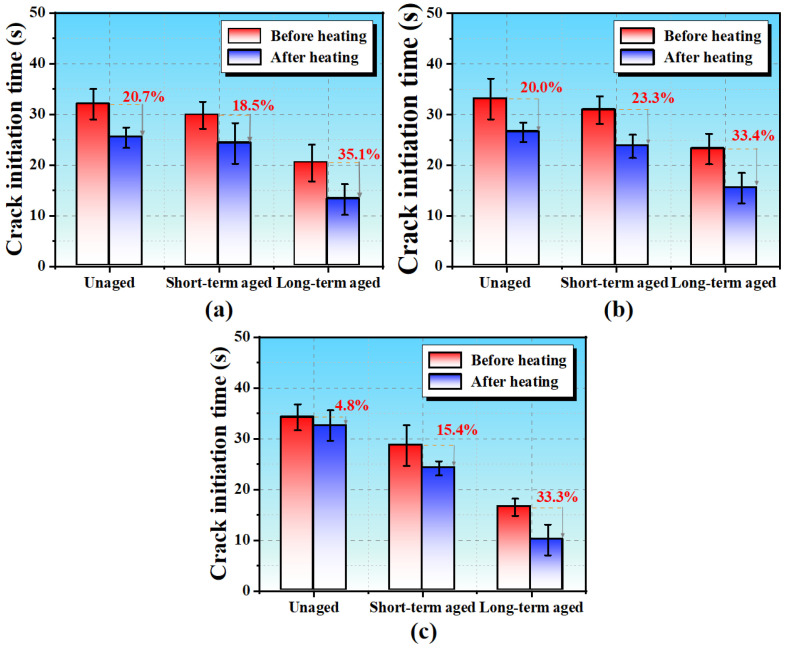
Crack initiation times of SSAM under different aging conditions: (**a**) AC-13; (**b**) SMA-13; (**c**) OGFC-13. Error bars indicate standard deviation (*n* = 3).

Figure 7 shows the crack initiation times for the three types of SSAM under different aging conditions. Aging significantly reduces crack initiation times and increases crack propagation rates, indicating decreased cracking resistance. For AC-13, the crack initiation time decreases from 32.0 s (unaged) to 20.41 s (long-term aged), and the propagation rate rises from 4.5 mm/s to 5.8 mm/s. SMA-13 shows a smaller reduction in initiation time, from 33.1 s to 23.1 s, and an increase in propagation rate from 7.5 mm/s to 9.3 mm/s, due to higher binder content, fiber reinforcement, and strong binder–aggregate adhesion. OGFC-13 is most sensitive, with initiation time dropping from 34.2 s to 16.5 s and propagation rate increasing from 5.1 mm/s to 6.4 mm/s; its high voids and thin binder film cause stress concentration and early cracking.

**Figure 8:**
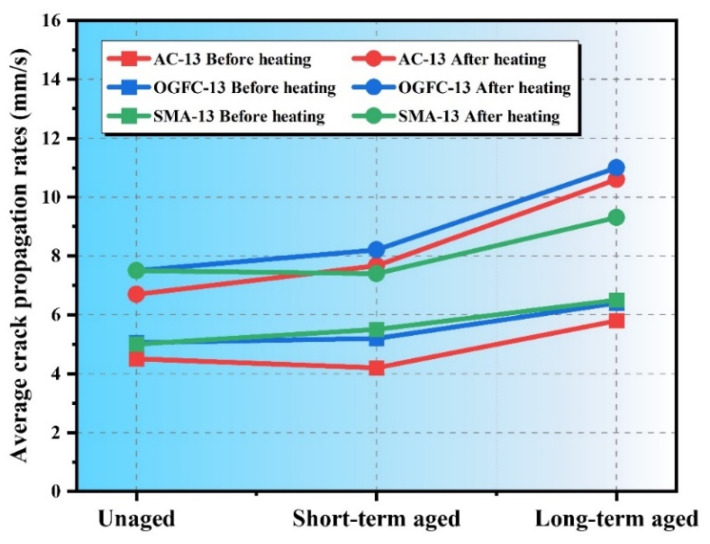
Average crack propagation rates of SSAM under different aging conditions.

Figure 8 shows the average crack propagation rates of SSAM under different aging conditions. The results demonstrate that both aging and microwave heating significantly affect the crack propagation rate, with varying effects on different mixture types. In the unaged state, the crack propagation rates of all mixtures were relatively low, with SMA-13 (5.1 mm/s) showing a slightly higher rate than AC-13 (4.5 mm/s) and OGFC-13 (5.0 mm/s). These results indicate that in the unaged condition, the mixture type has a minimal influence on crack propagation. After microwave heating, the crack propagation rate increased significantly for all mixtures, with AC-13 showing an increase of 2.1 mm/s, OGFC-13 an increase of 2.6 mm/s, and SMA-13 an increase of 2.4 mm/s. The effect of microwave heating was most pronounced on OGFC-13, likely due to its higher porosity, which facilitates the softening of the binder and promotes faster crack propagation. After short-term aging, the crack propagation rate increased for all mixtures, with AC-13 showing a notable increase from 4.3 mm/s to 7.5 mm/s, while SMA-13 only increased slightly (from 5.4 mm/s to 7.1 mm/s). This indicates that short-term aging has a more significant effect on AC-13 than on SMA-13. Microwave heating further increased the crack propagation rate for all mixtures, particularly for AC-13 and OGFC-13, where the increase was more pronounced (3.2 mm/s). This suggests that microwave heating significantly influences the crack resistance of short-term aged mixtures, especially for those with a more fragile structure such as AC-13 and OGFC-13. In the long-term aged mixtures, the crack propagation rate increased significantly for all mixtures, particularly for AC-13, which increased from 5.8 mm/s to 10.4 mm/s, and for OGFC-13, which increased from 6.5 mm/s to 11.1 mm/s. This demonstrates that long-term aging significantly weakens the structure of the mixtures, leading to a substantial reduction in crack resistance. After microwave heating, the crack propagation rate further increased for all long-term aged mixtures, with the highest increase observed in OGFC-13 (4.6 mm/s). These results indicate that while microwave heating partially restores binder flow and promotes self-healing, it also accelerates crack propagation due to the structural weaknesses induced by aging, especially in mixtures with higher porosity.

#### 3.2.3. Strain Field Analysis

By examining the strain fields at various time points, one can gain insights into the strain distribution and changes within SSAM under loading, which aids in assessing their load-bearing performance and self-healing capabilities [33]. The analysis of strain fields during three-point bending tests on beam specimens provides a comprehensive evaluation of the effects and mechanisms of microwave heating self-healing in aged mixtures, enhancing their performance and extending their service life.

**Figure 9:**
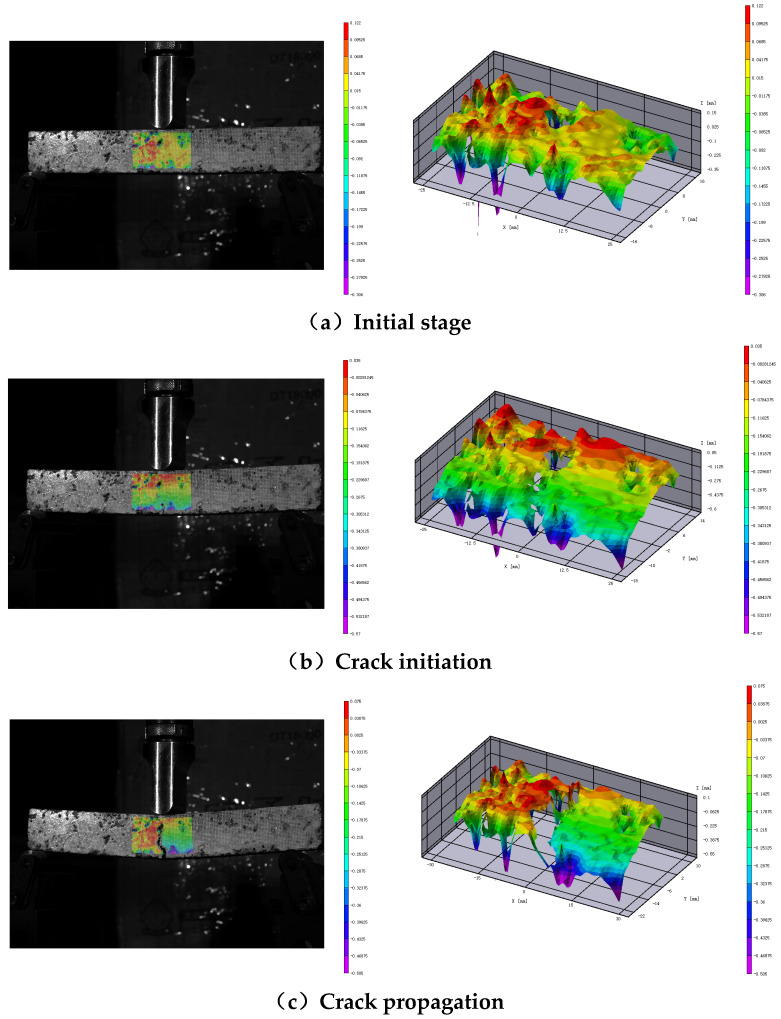
Evolution of crack displacement characteristics and 3D visualization.

**Figure 10:**
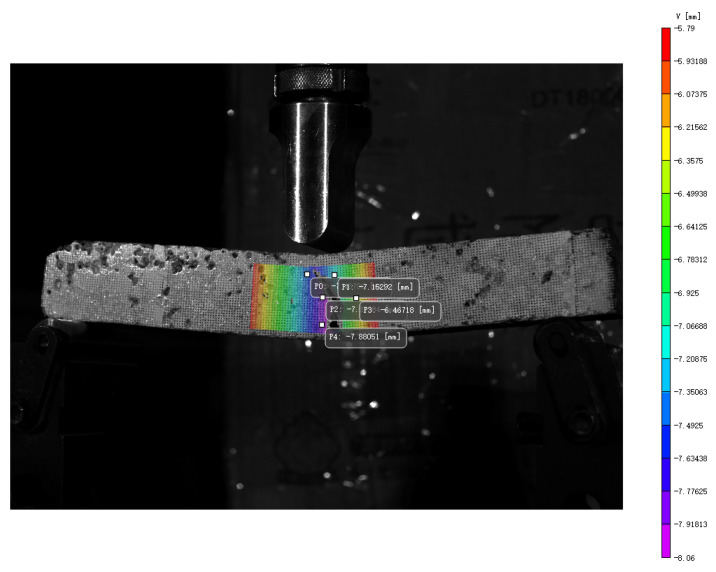
Surface sampling points on the specimen.

Figure 9 illustrates the displacement contours of asphalt mixture specimens at the stage of reflective cracking, indicating strain concentration due to tensile stresses at the mid-span. Therefore, this study selects a 4 cm × 3 cm region at the mid-span as the research area for crack damage expansion. The behavior of crack damage is evaluated by calculating the displacement and strain throughout the crack development process, as shown in Figure 10.

**Figure 11:**
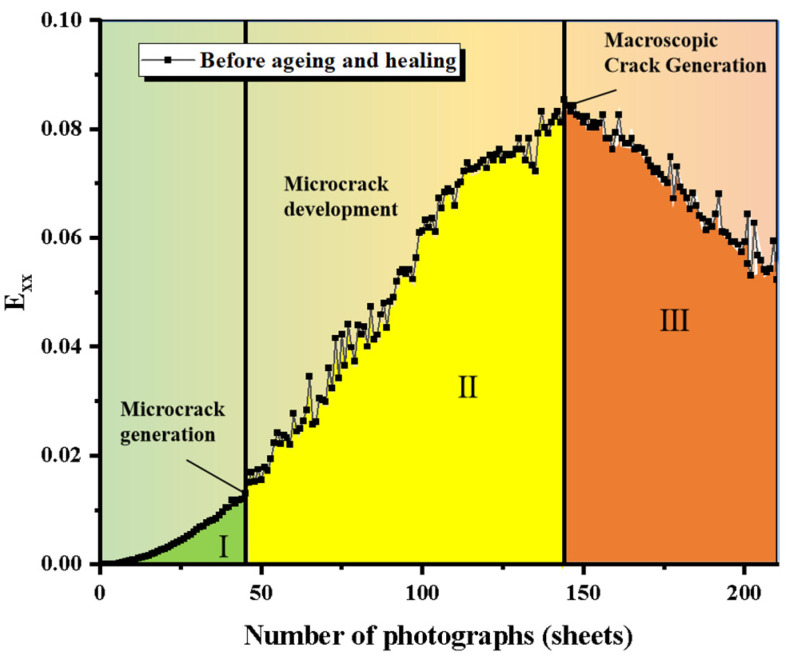
Horizontal strain (Exx) curve for unaged SSAM.

**Figure 12:**
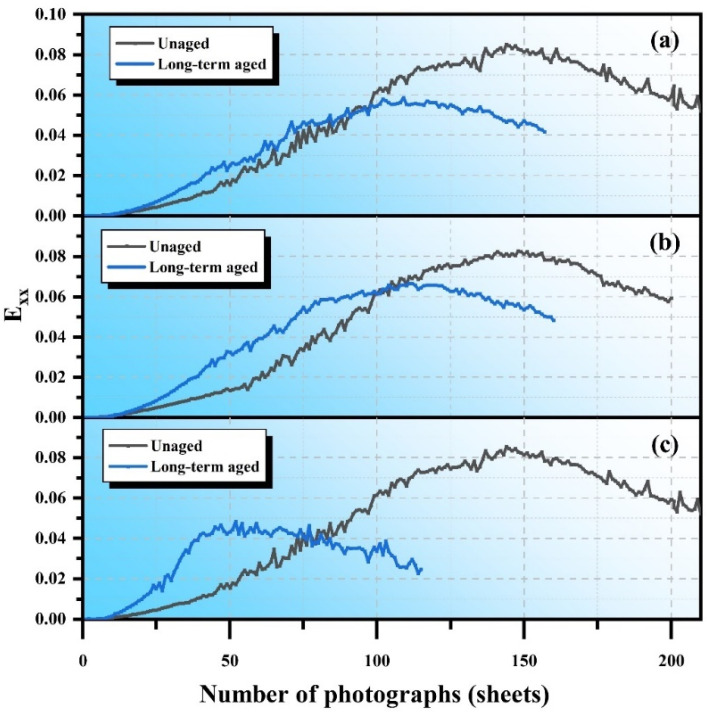
Horizontal strain (Exx) curves for SSAM after long-term aging: (**a**) AC-13; (**b**) SMA-13; (**c**) OGFC-13.

**Figure 13:**
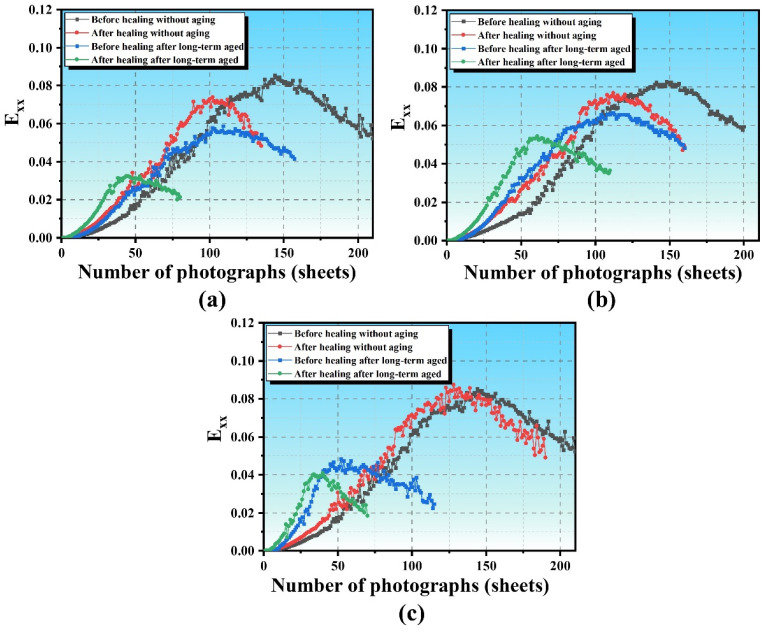
Horizontal strain (Exx) of SSAM after microwave heating: (**a**) AC-13; (**b**) SMA-13; (**c**) OGFC-13.

Long-term aging significantly impacts the performance of SSAM. Figure 11 illustrates the variation in *Exx* for unaged mixtures with the number of DIC images, revealing three main phases: stable strain growth, fluctuating strain growth, and strain reduction. Exx represents the normal strain in the horizontal direction obtained from DIC. Figure 12 shows the Exx curves for mixtures after long-term aging, indicating that as aging progresses, the crack resistance of the mixtures decreases, with unaged mixtures exhibiting higher strain values. Specifically, AC-13 decreases from 0.085 before aging to 0.058 after aging, a reduction of 31.0%; SMA-13 decreases from 0.082 to 0.066, a reduction of 19.5%; and OGFC-13 decreases from 0.085 to 0.048, a reduction of 43.4%. Figure 13 shows the Exx curve of mixtures after microwave heating self-healing, while the maximum horizontal strain values decrease, the healed areas remain fragile. The maximum strain values after healing for AC-13 and SMA-13 in their unaged states decrease by 13.5% and 6.9%, whereas OGFC-13 shows a slight increase. After long-term aging, the maximum horizontal strain values for AC-13, SMA-13, and OGFC-13 decrease by 44.8%, 18.5%, and 13.1%, indicating that aging reduces the healing capacity of the mixtures. Aging causes the asphalt to harden and become brittle, thereby limiting its self-healing effectiveness.

## 4. Conclusions

This study systematically evaluated the microwave heating self-healing performance of asphalt and steel slag asphalt mixtures (SSAMs) before and after short- and long-term aging using DSR, BBT, and DIC techniques. The main findings are as follows:

1. The healing index of asphalt increases with rest time, reaching approximately 70.4% for unaged asphalt, decreasing to 58.1% after short-term aging and 43.0% after long-term aging. The fitted healing curves have high R2 values (>0.87), confirming the reliability of the measurements.
2. Aging affects SSAM differently. Generally, aging reduces load-bearing capacity and tensile strength while increasing stiffness. Long-term aging has the most pronounced impact, with OGFC-13 being the most affected, AC-13 intermediate, and SMA-13 least sensitive.
3. Crack initiation times decrease after aging, accompanied by reduced strain values and forward-shifted peak horizontal strains. SMA-13 exhibits the smallest reduction in peak strain, indicating lower sensitivity to aging.
4. The microwave self-healing rate declines with increasing aging. For AC-13, the rate drops from 60% (unaged) to 46.9% (long-term aged); for OGFC-13, from 71% to 52.4%; and for SMA-13, from 57.4% to 45.3%.
5. After microwave healing, the crack initiation time shortens and the crack propagation rate increases. Maximum horizontal strains decrease with aging, showing reductions of 0.026, 0.012, and 0.007 for AC-13, SMA-13, and OGFC-13, respectively, highlighting increased brittleness after long-term aging.
6. The observed reduction in healing indices indicates that microwave repair effectiveness diminishes over time. To maintain pavement performance, mixtures with higher intrinsic healing potential or additives enhancing binder flow and adhesion can be used. Furthermore, understanding predicted aging levels can guide the selection of mixture types and repair strategies for different pavement sections.

## Figures and Tables

**Table 1:** Main performance indicators of materials.

Materials	Indicators		Values	Units
Asphalt	Density		1.03	15 °C, g/cm^3^
	Penetration		70	25 °C, 0.1 mm
	Softening Point		48	°C
	Ductility		>100	15 °C, mm
Coarse aggregate	Apparent Relative Density		2.73	-
	Crushing Value		20.90	%
	Water Absorption		1.06	%
Fine aggregate	Apparent Relative Density		2.74	-
	Clay Content		2.30	%
	Sand Equivalent		68	%
Filler	Apparent Relative Density		2.75	-
	Particle Size Range	<0.6 mm	100	%
		<0.15 mm	99.4	%
		<0.075 mm	88.0	%
	Hydrophilic Coefficient		0.7	-
	Appearance		No Clumping	-
SSA	Apparent Relative Density		3.41	-
	Los Angeles Wear		12.72	%
	Crushing Value		15.41	%
	Polishing Value		56	%
	Water Absorption		1.94	%
	Free Calcium Oxide Content		1.14	%

**Table 2:** Chemical composition of SSA (%).

CaO	Fe_2_O_3_	S_i_O_2_	M_g_O	Al_2_O_3_	f-CaO	SO_3_
44.18	21.54	13.69	5.92	2.76	1.81	negligible

**Table 3:** Healing models and fitting parameters for asphalt under different aging conditions.

Aging Condition	Healing Model	R^2^	R_0_	K
Unaged	H(t) = 0.2082 + 0.2862t^1/4^	0.9387	0.2082	0.2862
Short-Term Aged	H(t) = 0.1718 + 0.2363t^1/4^	0.9025	0.1718	0.2363
Long-Term Aged	H(t) = 0.1438 + 0.1649t^1/4^	0.8760	0.1438	0.1649

**Table 4:** Results of three-point bending tests for unaged SSAM before and after microwave heating.

Test Results	AC-13		OGFC-13		SMA-13	
	Unaged	After Heating	Unaged	After Heating	Unaged	After Heating
Maximum Load (kN)	1.05	0.63	1.17	0.83	1.26	0.72
Mid-span Deflection (mm)	0.611	0.711	0.621	0.531	0.733	0.641
Bending Strength (MPa)	8.3	5.1	8.9	6.7	10.2	6.3
Stiffness Modulus (MPa)	2670.2	1378.8	2929.7	2437.2	2649.3	1889.4
Bending Strain (10^−3^)	3.21	3.73	3.26	2.78	3.85	3.37

**Table 5:** Results of three-point bending tests for short-term aged SSAM before and after microwave heating.

Test Results	AC-13		OGFC-13		SMA-13	
	Short-Term Aged	After Heating	Short-Term Aged	After Heating	Short-Term Aged	After Heating
Maximum Load (kN)	0.99	0.56	1.20	0.79	1.32	0.71
Mid-span Deflection (mm)	0.524	0.613	0.497	0.577	0.644	0.617
Bending Strength (MPa)	8.1	4.5	9.8	6.4	9.3	5.7
Stiffness Modulus (MPa)	2938.7	1419.7	3753.2	2128.3	3188.0	1788.8
Bending Strain (10^−3^)	2.75	3.22	2.61	3.03	3.38	3.24

**Table 6:** Results of three-point bending tests for long-term aged SSAM before and after microwave heating.

Test Results	AC-13		OGFC-13		SMA-13	
	Long-Term Aged	After Heating	Long-Term Aged	After Heating	Long-Term Aged	After Heating
Maximum Load (kN)	0.54	0.25	0.52	0.27	0.77	0.34
Mid-span Deflection (mm)	0.433	0.396	0.621	0.531	0.733	0.641
Bending Strength (MPa)	4.4	2.3	4.2	2.2	6.4	2.7
Stiffness Modulus (MPa)	2999.3	1742.4	4038.4	2340.7	3950.4	2410.5
Bending Strain (10^−3^)	1.47	1.32	1.04	0.94	1.62	1.12

## Data Availability

The original contributions presented in this study are included in the article/Supplementary Material. Further inquiries can be directed to the corresponding authors.

## Supplementary Materials

The following supporting information can be downloaded at: https://www.mdpi.com/article/10.3390/polym17192678/s1, Table S1: Complex shear modulus of unaged asphalt at different rest periods. Table S2: Complex shear modulus of short-term aged asphalt at different rest periods. Table S3: Complex shear modulus of long-term aged asphalt at different rest periods.
