# Supplementary material for: Effects of Aged Conditions on the Self-Healing Performance of Asphalt Mixtures: A Comparative Study of Long-Term and Short-Term Aging
Source: Polymers (Basel). 2025 Oct 3;17(19):2678. doi: 10.3390/polym17192678 (PMC12526552; doi:10.3390/polym17192678)
Supplement: Supplementary file 1 [file polymers-17-02678-s001.zip › polymers-3854498-supplementary.pdf]

Supplementary Material

Table S1. Complex shear modulus of unaged asphalt at different rest periods.

Rest Period (s)	G_i^* (MPa)	G_L^* (MPa)	G_r^* (MPa)	Healing Index
5	6.52	3.91	4.35	0.17
10	6.78	4.06	5.17	0.41
15	6.81	4.22	5.33	0.43
30	6.54	3.98	5.15	0.46
60	6.64	4.05	5.37	0.51
120	6.73	4.03	5.59	0.58
300	6.55	3.99	5.83	0.72
600	6.47	3.88	5.92	0.79
900	6.86	4.18	6.40	0.83
1800	6.78	4.20	6.44	0.87
2700	6.77	4.06	6.55	0.92
3600	6.44	3.92	6.44	1.00
5400	6.81	4.08	6.97	1.06
7200	6.72	4.09	7.00	1.11

Table S2. Complex shear modulus of short-term aged asphalt at different rest periods.

Rest Period (s)	G_i^* (MPa)	G_L^* (MPa)	G_r^* (MPa)	Healing Index
5	10.20	6.22	6.49	0.07
10	9.67	5.99	7.35	0.37
15	9.89	5.93	7.51	0.40
30	10.08	6.04	7.69	0.41
60	9.57	5.83	7.55	0.46
120	9.49	5.88	7.64	0.49
300	9.87	5.92	8.05	0.54
600	9.62	5.96	8.15	0.60
900	10.23	6.13	8.75	0.64
1800	9.21	5.61	8.27	0.74
2700	9.43	5.65	8.63	0.79
3600	9.65	5.79	8.99	0.83
5400	9.33	5.59	8.84	0.87
7200	10.24	6.14	9.95	0.93

Table S3. Complex shear modulus of long-term aged asphalt at different rest periods.

Rest Period (s)	G_i^* (MPa)	G_L^* (MPa)	G_r^* (MPa)	Healing Index
5	22.44	13.46	14.26	0.09
10	23.52	14.34	16.26	0.21
15	23.46	14.07	16.32	0.24
30	22.79	13.90	16.83	0.33
60	22.41	13.45	16.76	0.37
120	23.51	14.11	17.68	0.38
300	23.78	14.50	18.49	0.43
600	22.01	13.43	17.46	0.47

900	23.09	13.85	18.56	0.51
1800	22.88	13.72	18.75	0.55
2700	22.37	13.64	18.87	0.60
3600	23.47	14.55	19.93	0.60
5400	23.56	14.84	20.13	0.61
7200	24.33	14.83	20.74	0.62
